# Farnesyltransferase inhibitor and rapamycin correct aberrant genome organisation and decrease DNA damage respectively, in Hutchinson–Gilford progeria syndrome fibroblasts

**DOI:** 10.1007/s10522-018-9758-4

**Published:** 2018-06-15

**Authors:** Mehmet U. Bikkul, Craig S. Clements, Lauren S. Godwin, Martin W. Goldberg, Ian R. Kill, Joanna M. Bridger

**Affiliations:** 1 Progeria Research Team, Healthy Ageing Theme, Institute for Environment, Health and Societies, College of Health and Life Sciences, Brunel University London, Kingston Lane, Uxbridge, UB8 3PH UK; 2 Department of Biosciences, Durham University, Science Laboratories, South Road, Durham, DH1 3LE UK

**Keywords:** Hutchinson–Gilford progeria syndrome, Genome organisation, COMET assay, DNA damage, Farnesyltransferase inhibitors, Rapamycin

## Abstract

Hutchinson–Gilford progeria syndrome (HGPS) is a rare and fatal premature ageing disease in children. HGPS is one of several progeroid syndromes caused by mutations in the *LMNA* gene encoding the nuclear structural proteins lamins A and C. In classic HGPS the mutation G608G leads to the formation of a toxic lamin A protein called progerin. During post-translational processing progerin remains farnesylated owing to the mutation interfering with a step whereby the farnesyl moiety is removed by the enzyme ZMPSTE24. Permanent farnesylation of progerin is thought to be responsible for the proteins toxicity. Farnesyl is generated through the mevalonate pathway and three drugs that interfere with this pathway and hence the farnesylation of proteins have been administered to HGPS children in clinical trials. These are a farnesyltransferase inhibitor (FTI), statin and a bisphosphonate. Further experimental studies have revealed that other drugs such as *N*-acetyl cysteine, rapamycin and IGF-1 may be of use in treating HGPS through other pathways. We have shown previously that FTIs restore chromosome positioning in interphase HGPS nuclei. Mis-localisation of chromosomes could affect the cells ability to regulate proper genome function. Using nine different drug treatments representing drug regimes in the clinic we have shown that combinatorial treatments containing FTIs are most effective in restoring specific chromosome positioning towards the nuclear periphery and in tethering telomeres to the nucleoskeleton. On the other hand, rapamycin was found to be detrimental to telomere tethering, it was, nonetheless, the most effective at inducing DNA damage repair, as revealed by COMET analyses.

**Electronic supplementary material:**

The online version of this article (10.1007/s10522-018-9758-4) contains supplementary material, which is available to authorized users.

## Introduction

### Genome organisation

Cell nuclei are the organelles that house an organisms’ genome. The chromosomes that make-up the genome are organised very specifically, with individual chromosomes located within their own individual space, hence the term “chromosome territories”. Given all the processes the chromosomes have to go through, such as replication, transcription and repair, it is predictable that a system by which chromatin avoids being tangled up in the nucleus would evolve. This is regulated organisation at the intrachromosomal level but the cell takes this type of spatial organisation further to the interchromosomal level by regulating the specific locations of individual chromosomes radially with respect to the nuclear edge. In proliferating cells gene-poor chromosomes are found to be located at the nuclear periphery with gene-rich chromosomes within the nuclear interior (Boyle et al. 2001; Bridger et al. 2014). The gene-density distribution of chromosomes is found in proliferating primary cells but when cells come out of the cell cycle into quiescence or senescence chromosomes are rearranged into a size-correlated distribution with large chromosomes at the nuclear periphery and small chromosomes in the nuclear interior (Bridger et al. 2000; Mehta et al. 2010a).

The reason for this spatial control over chromosome territories is not yet entirely clear but it is postulated to regulate the function of the genome through position and association of different chromatin types with nuclear structures, thus controlling gene expression (Lemaître and Bickmore 2015). How does this specific positioning come about? There are a number of structures which provide scaffolding to the genome and anchor chromatin at specific points around the nucleus through chromatin binding interactions with specific proteins. These include the nuclear lamina, a mesh-like structure subjacent to the inner nuclear envelope comprised of A-type and B-type lamins, the nuclear membrane and proteins found therein, the nucleolus and other structures found throughout the nucleoplasm, termed the nucleoskeleton (Bourne et al. 2013; Czapiewski et al. 2016). A-type lamins are involved in many processes including those associated with genome regulation and function. We know for example that lamin A is important in the correct positioning of chromosome territories in interphase nuclei (Meaburn et al. 2007; Ondrej et al. 2008; Mehta et al. 2011; Puckelwartz et al. 2011; Harr et al. 2015), anchoring them at the nuclear periphery (Kubben et al. 2012; Solovei et al. 2013; Kind and van Steensel 2014) or throughout the nucleus bound to a nucleoskeleton containing lamin A (Bridger et al. 1993; Hozak et al. 1995; Broers et al. 1999; Neri et al. 1999; Elcock and Bridger 2008; Mehta et al. 2008; Barboro et al. 2010). Telomeres seem to be one of the tethering points for the genome since telomeric repeats and sequences bind to lamin A (Shoeman and Traub 1990; Barboro et al. 2003; Ottaviani et al. 2009; De Vos et al. 2010; Uhlírová et al. 2010; Das et al. 2013; Saha et al. 2013; Wood et al. 2014) and interaction of telomeres with defective lamin A can cause cellular senescence (Cao et al. 2011a). Luderus and colleagues have postulated that matrix attachment regions (MARs) show an affinity to tether specific areas of mammalian telomeres to a nucleoskeleton and have suggested that per kb of telomeric sequence, that at least one MAR is present (Luderus et al. 1996). It has been demonstrated that human telomeres are tethered to the nuclear membrane by their TTAGGG repeats (de Lange 1992).

Using the genome-wide mapping of protein-DNA interactions it has been revealed that gene-poor regions of the genome do indeed interact with the nuclear lamin proteins present at the nuclear periphery (Peric-Hupkes and van Steensel 2010; Guelen et al. 2008). These regions have been termed lamina associated domains (LADs). Indeed, these experiments finally confirm what has been believed for many decades that nuclear lamin proteins anchor the genome at the nuclear periphery. However, lamin proteins are not only found at the periphery of nuclei but are also found throughout the nucleoplasm in small accumulations (Naetar et al. 2017). The structures that internal lamin are part of are poorly defined compared to their well-studied organisation at the nuclear envelope, but they are described as being a part of an internal matrix or nucleoskeleton (Bridger et al. 2007).

### Hutchinson–Gilford progeria syndrome: a laminopathy

The premature ageing syndrome Hutchinson–Gilford progeria syndrome (HGPS) is a severe childhood disease that leads to an early death usually from heart attacks and strokes as a teenager (Ahmed et al. 2017). HGPS is normally caused by the silent mutation G608G in the *LMNA* gene, encoding for the A-type lamins—nuclear lamins A and C. This mutation creates a splice variant leading to a new protein, progerin, which is a 50 amino acid truncated version of lamin A. This protein cannot be processed correctly. Progerin is toxic to cells since it retains a farnesyl group which affects its incorporation into the nuclear envelope and ultimately its functionality. Progerin negatively affects the disassembly of the nuclear envelope at the beginning of mitosis as it remains with the membrane material rather than being released and becoming soluble during mitosis (Wu et al. 2014). Since A-type lamins have been described as chromatin binding proteins HGPS and other laminopathies, have become a model to study genome organisation and the involvement of lamin proteins. Indeed, we have demonstrated, painting whole chromosomes in interphase nuclei, that genome organisation and chromosome positioning are affected in classic HGPS fibroblasts such that chromosomes normally found at the nuclear periphery in normal proliferating fibroblasts are now found towards the nuclear interior (Meaburn et al. 2005; Meaburn et al. 2007; Mehta et al. 2011). Further, studies assessing the chromosomal sequence attachment at the nuclear lamina also show alterations in genome organisation when compared to normal cells (Kubben et al. 2012).

We initially proposed that the lamin A mutation must have affected the anchorage of the gene-poor chromosomes with progerin preventing them attaching to the peripheral nuclear lamina and therefore finding docking elsewhere in the nucleus. However, this interpretation is too simple because chromosomes such as the X chromosome territories are still in position at the nuclear edge. It was then thought that these cells mirrored senescent cells with respect to chromosome positioning since they are derived from patients with premature ageing syndrome. When the position of chromosome 10 was analysed it became clear that the HGPS cells, even though they are proliferating, have a signature of chromosome location common to quiescent human dermal fibroblasts. This is because chromosome 10 has a peripheral location in quiescent primary fibroblast nuclei and not in senescent cells, implying that genome organisation is not similar to aged senescent cells but pathways linking proliferation with lamins have become disconnected or unregulated (Mehta et al. 2011; Bridger et al. 2014). The association of *LMNA* mutations with altered chromosome territories was shown in *LMNA* mutant fibroblasts as chromosome 13 was displaced to the nuclear interior in *LMNA* E161 K mutants cells (Mewborn et al. 2010), whereas chromosome 13 was shown positioning against the nuclear membrane in *LMNA* D596 N mutant cells (Puckelwartz et al. 2011) and in S143F and E145 K mutations of *LMNA* (Bercht Pfleghaar et al. 2015; Taimen et al. 2009, respectively). Most telomeres avoid the nuclear periphery and are dispersed throughout the nuclear interior, suggesting a more specific role for intranuclear lamins in tethering telomeres (Shimi et al. 2008). A repositioning of telomere localization was observed towards the nuclear periphery in cells lacking A-type lamins, suggesting an active role of A-type lamins in the positioning of telomeres in the nuclear interior (Gonzalez-Suarez et al. 2009).This reveals two things about HGPS—that the progerin with its uncleaved farnesyl group has uncoupled chromosome behaviour from the cell cycle and that HGPS has nuclei that resemble quiescent rather than senescent cells (Bridger et al. 2014).

### Drug treatments for HGPS

There are now a number of drugs that have been investigated as treatments for HGPS both in the clinic and in the laboratory. These include farnesyl transferase inhibitors (FTIs) that block the transfer of the farnesyl group rendering a truncated lamin A protein without the farnesyl groups. Subsequently, other drugs that work in the mevalonate pathway the statin—pravastatin and the bisphosphonate zoledronic acid were investigated. Drug trials were conducted in the US that were purely FTI—Lonafarnib initially (Gordon et al. 2012) and another in Europe where pravastatin and zoledronic acid were used in combination (Blondel et al. 2014). A second clinical trial in the US was initiated using FTI, plus statin and the bisphosphonate (Gordon et al. 2014). Further investigations in a number of laboratories have suggested other drugs/treatments that would/could treat HGPS—these include the *N*-acetyl cysteine, free radical scavenger (Richards et al. 2011), rapamycin—the new anti-ageing treatment (Cao et al. 2011b; Cenni et al. 2012; Graziotto et al. 2016; Evangelisti et al. 2016), and IGF-1 (Mariño et al. 2010).

In the past we have performed genome organisation studies with FTI with and without geranylgeranyltransferase inhibitors (GGTI) (required as cells can compensate for the lack of farnesylation with geranylation). Using FTI singularly and in combination with GGTI we have revealed that these drugs do indeed correct chromosome positioning—allowing gene-poor chromosomes such as chromosomes 13 and 18 to be located at the nuclear envelope in proliferating HGPS cells (Mehta et al. 2011). Implying that the farnesyl group itself is causing the uncoupling of pathways. However, we have not tested any of the other employed or potential drugs on genome organisation until now. Furthermore, we have gone further than before and analysed other aspects of genome intra-nuclear organisation for nine different drug regimes. Using HGPS cells with the classical G608G mutation we have analysed the effect of specific drugs on the HGPS phenotype including chromosome territory positioning, telomeric attachment to the nucleoskeleton in DNA Halo preparations and the extent of DNA damage and repair markers through immunofluorescence staining and COMET analysis.

## Materials and methods

### Cell culture

An HGPS cell-line derived from AG01972 (14 years old female primary HGPS) was obtained from Coriell Cell Repositories (New Jersey, USA) at the earliest passage available and passaged twice weekly at a specified density. This HGPS cell-line proliferated well, with a Ki67 index > 50% initially. Samples were taken for all assays at similar times. The 2DD normal dermal fibroblast cell line was used as a control at passage numbers between 7 and 15 (Bridger et al. 1993). This line has a normal karyotype. These cell lines were cultured in T75 tissue culture flasks (Fisher, UK) in Dulbecco’s Modified Eagles Medium (DMEM) (Invitrogen, UK), with the following additives: 15% foetal bovine serum (FBS) (Invitrogen), 2% (v/v) streptomycin and penicillin antibiotics (Invitrogen) and 200 mM l-glutamine (Invitrogen). The cells were maintained at 37 °C temperature, in a humidified atmosphere containing 5% CO_2._ Cells were washed with 3 ml of versene (NaCl 0.8% (w/v), KCl 0.02% (w/v), Na2HPO4 0.0115% (w/v), KH2PO4 0.02% (w/v), 0.2% EDTA (w/v) (Sigma Aldrich)). Followed by 3 ml diluted solution of 0.25% trypsin (Invitrogen): versene (1:10, v: v) for up to five minutes. As soon as all the cells were detached from the flasks’ bottoms, the effect of trypsin solution was neutralized by adding an equal amount of DMEM medium to the dishes. The final suspension was centrifuged at 300–400 g for 5 min. The cells were then seeded in T75 flasks at a density of 5 × 10^5^/flask.

### Drug treatments

The drugs employed in this study were FTI-277, Pravastatin, Zoledronic acid, Rapamycin, Insulin-like growth factor 1, *N*-acetyl-l-cysteine and GGTI-2133 and were all obtained from Sigma Aldrich, UK. Drugs were added to the media and incubated for fixed periods of time. The final concentration and duration of drug treatments were: FTI-277—2.5 µM for 48 h (Mehta et al. 2011), pravastatin—1 µM for 24 h (Varela et al. 2008), zoledronic acid—1 µM for 24 h, rapamycin—10 nM for 24 h (Cao et al. 2011b), insulin-like growth factor 1—50.0 ng/mL for 24 h (Mariño et al. 2010), *N*-acetyl-l-cysteine—20 µM for 1 h (Richards et al. 2011), FTI-277 and GGTI-2133—both 2.5 µM for 48 h) (Mehta et al. 2011; Kieran et al. 2007), pravastatin and zoledronic acid—both 1 µM for 24 h (Varela et al. 2008) and FTI-277—2.5 µM for 48 h, pravastatin and zoledronic acid—both 1 µM for 24 h.

### Two-dimensional fluorescence in situ hybridisation

Cells were harvested by incubation 0.25% trypsin in versene and centrifuged at 300–400 g for 5 min, and then the supernatant was removed. Harvested cells were treated with a hypotonic solution (0.075 M KCl) for 15 min at room temperature, to swell the cells. The cell suspension was centrifuged at 300 g for 5 min using a bench top centrifuge. The cells were fixed by removing the supernatant and re-suspending the cell pellet in ice-cold methanol: acetic acid (3:1 v/v), added drop-wise to the cells with gentle agitation. The cells were incubated at 4 °C for at least 1 h and then the fixation step was repeated 4-5 more times before storage or − 20 °C. The cells were then dropped onto damp SuperFrost™ slides from a height, and air dried and aged at 70 °C for 1 h or for 2 days at room temperature. The aged slides were passed through an ethanol row of 70, 90 and 100% ethanol for 5 min in each solution, followed by air drying. The slides were then incubated in denaturation solution (70% {v/v} formamide, 2X SSC, pH 7.0) at 70 °C for 5 min. The slides were immediately plunged into ice-cold 70% ethanol for 5 min and then again passed through the ethanol row (90 and 100% ethanol). The slides were air dried and kept warm until hybridization with the probe.

The chromosome templates 18 and X were made in-house by amplifying flow sorted chromosomes by degenerate oligonucleotide primed polymerase chain reaction (DOP-PCR). These chromosome templates were labelled with biotin-16-dUTP (Roche). The slides were allowed to hybridise with the probe in a humid chamber at 37 °C for at least 18 h, washed and mounted in Vectashield (Vectalabs) mounting medium containing DAPI.

### Indirect immunofluorescence

In order to distinguish proliferating cells from non-proliferating cells, 2D-FISH was combined with indirect immunofluorescence staining for antigen pKi-67. PKi-67 is a nucleolar antigen that is only present in cells in proliferative cell cycle (Gerdes et al. 1983). The coverslips from the FISH experiments were removed by placing slides in 1× PBS solution for 30 min on a shaker until the coverslips detach. The slides were incubated 100 µl of rabbit primary anti-pKi-67 (DAKO, A0047) (1:30 dilution in 1% NCS/PBS v/v) and placed in an humidified chamber either for 2–3 h at room temperature or overnight at 4 °C, followed by 27 washes in 1× PBS. Subsequently, fluorochrome-conjugated secondary antibody, the swine anti-rabbit (TRITC) antibody (DAKO, R0156) diluted with 1% FBS in 1× PBS was added to the slides. Cells were incubated in the dark for either 30 min at 37 °C or 1 h at room temperature. The cells were washed 27 times in 1× PBS followed by a final wash in double distilled water. Mouse monoclonal anti-human lamin A/C (Novocastra) diluted 1:10 with FITC-conjugated secondary antibody (diluted 1:30 (Dako), was used to reveal A-type lamins and Vectashield containing DAPI (Vector Laboratories) was added to each slide and the coverslip.

For the γH2AX DNA damage assay, cells were washed 15 min two times with PBS. 100 μl of γ-H2AX antibody (dilution of 1:500 with 1% PBS/FCS, Upstate) was added to the slides for 1 h at room temperature then after which slides were washed in PBS for 15 min. 100 µl of goat anti-mouse (FITC) (diluted 1:64 with 1% PBS/FCS, Sigma-F9006) secondary antibody was added for 1 h at room temperature and then rinsed with PBS for 15 min and mounted in Vectashield.

### DNA halo preparations

Cells were grown for 2 days on SuperFrost^®^ slides (Fisher, UK) in QuadriPERM™ (Sarstedt, Germany) chambers, at a starting density of 1 × 10^5^. The medium was removed and the slides were washed 3 times with 1× PBS. Slides were then placed in Coplin jars, on ice, containing CSK buffer (10 mM Pipes pH 7.8, 100 mM NaCl, 0.3 M sucrose, 3 mM MgCl_2_, 0.5% (v/v) Triton-X 100) for 15 min. The slides were then washed again three times in 1× PBS. The slides were placed in extraction buffer (2 M NaCl, 10 mM Pipes pH 6.8, 10 mM EDTA, 0.1% (w/v) digitonin, 0.05 mM (v/v) spermine, and 0.125 mM (v/v) spermidine) for 4 min. The slides were then rinsed consecutively in 10× PBS, 5× PBS, 2× PBS and 1× PBS, for 1 min each. The slides were taken through an ethanol series of 10, 30, 70 and 95% (v/v), respectively. The slides were air dried and stored. Fifty images of cell nuclei for each condition were taken at ×100 magnification using a Zeiss Axiovert 200 M with a Plan-NEOFLUAR ×100/1.30 objective lens (Carl Zeiss, Germany) and high resolution digital camera (AxioCam b/w, Carl Zeiss, Germany) under the control of Axiovision software (Carl Zeiss). Measurements for area of residual nucleus and extent of halo were measured with ImageJ software, and the ratio of the DNA halo to residual nucleus was calculated and plotted (Elcock and Bridger 2010; Clements et al. 2016).

For bromo-deoxy uridine incorporation into the genome in order to visualise proliferating cells, the medium was removed and replaced with medium containing BrdU and FrdU (3 µg/µl) (Sigma Aldrich). After 24 h, the media was removed, cells were washed once with medium (10% NCS) and then re-fed with medium (10% NCS). Following an additional 24 h, the slides were taken through the DNA halo preparation. Slides were washed 3 times in PBS and incubated at room temperature for 1 h in BrdU mouse anti-human diluted 1:100 (BD Pharmingen). Cells were then washed 3 times in PBS and incubated at room temperature for 1 h in donkey anti-mouse Cy3 diluted 1:100 (Jackson Laboratory). Following 3 more washes with PBS, slides were mounted with Vectashield (Vector Laboratories) containing DAPI.

### Telomere PNA FISH

In order to detect telomeres on the DNA halo preparations, a Telomere PNA FISH/Cy3 kit (Dako) was used following a protocol under the manufacturer’s instructions. Unless otherwise stated, the protocol was performed at room temperature. The DNA halo slides were immersed in tris-buffered saline (TBS, pH 7.5) for 2 min in 3.7% formaldehyde (in TBS, v/v). The slides were then washed in TBS twice for 5 min each. They were then immersed in pre-treatment solution for 10 min and subsequently washed twice with TBS for 5 min. The slides were then run through an ice cold ethanol series consisting of 70, 85 and 95% (v/v) ethanol for 2 min at each concentration. Slides were air dried and 10 µl of Telomere PNA Probe/Cy3 was added to each slide. The coverslip was replaced and then the slides were placed in a pre-heated oven at 80 °C for 5 min and then in the dark for approximately 1 h. The coverslips were removed by immersing the slides in the Rinse Solution for 1 min and then in Wash Solution for 5 min at 65 °C. The slides were subjected to a further ice-cold ethanol series (70, 85 and 95% (v/v)) for two minutes at each concentration, and then air dried. Slides were mounted with Vectashield (Vector Laboratories) containing DAPI. Images were captured at ×100 magnification using a Zeiss Axiovert 200M with a Plan-NEOFLUAR ×100/1.30 objective lens (Carl Zeiss, Germany) and high resolution digital camera (AxioCam b/w, Carl Zeiss, Germany) under the control of Axiovision software (Carl Zeiss). The proportions of telomeres within residual nuclei for each treatment were counted and expressed as a percentage of the total telomeres.

### Microscopy and data analysis

For chromosome positioning analyses slides were examined using a Zeiss Axiovert 200M with a Plan-NEOFLUAR ×100/1.30 objective lens (Carl Zeiss, Germany) and high resolution digital camera (AxioCam b/w, Carl Zeiss, Germany) under the control of AxioVision software (Carl Zeiss). Nuclei were selected randomly by following a rectangular scan pattern, and grey-scale images of these nuclei with the DAPI signal pseudo-coloured in blue and biotin-streptavidin signal pseudo-coloured in green were captured. At least 50–60 images per slide were taken and converted into TIFF or PICT format. The images were passed through a bespoke erosion analysis script using IPLab Spectrum software (a kind gift of Prof Wendy Bickmore, Edinburgh). Similar scripts have been devised and can be found in the literature from the laboratories of Profs Eric Schirmer, BJ Rao and Christopher Eskiw (Robson et al. 2016; Mehta et al. 2013; Gillespie et al. 2015). The script is devised to divide each captured nucleus into 5 concentric shells of equal area, the first shell starting at the periphery of the nucleus going to the interior of the nucleus (5th shell). The script measures the pixel intensity of DAPI and the chromosome probe in the five shells. The background from the FISH signal was removed by subtracting the mean pixel intensity within the segmented nucleus. The probe signal was normalised by dividing the percentage of the probe by the percentage of DAPI signal in each shell. The normalized proportion of probe was calculated in all 5 shells for at least 50 nuclei (Croft et al. 1999; Clements et al. 2016). The data were plotted as bar charts.

### Statistical analysis

Values are expressed as averages ± SEM, and n represents the number of experiments analysed. Individual treatments were compared using either two-tailed unpaired t-tests or one-way ANOVAs with Tukey’s post hoc multiple comparison tests where appropriate, and significance was taken as P ≤ 0.05. The level of significance is indicated as: *  P  ≤ 0.05, **P ≤ 0.01, ***P ≤ 0.001 and ****P ≤ 0.0001. Analysis was performed using Graphpad Prism 6.0 for Windows package.

### Comet assay

To estimate endogenous levels of DNA damage in treated and untreated progeria cells, the single cell gel electrophoresis (alkaline comet) assay was used. Comet assays were performed using an Fpg FLARE™ Assay Kit according to the manufacturer’s instructions (Trevigen). Prepared slides were placed in a cooled electrophoresis tank and electrophoresis was performed at 1 V/cm for 30 min. Slides were washed twice in dH_2_O for 5 min each, then in 70% ethanol for 5 min before air drying to bring all cells into a single focal plane. SYBR^®^ Green was added to each sample and incubated at room temperature in the dark for 30 min. Excess solution is removed and the slides again left to air-dry completely. Slides were viewed and analysed with a 20× objective mounted on a Zeiss Axioplan2 microscope controlled by the Metafer MetaCyte cometscan software. Estimates of DNA damage level in each cell were determined using Comet tail moments (% of DNA in tail × tail length in μm) of at least 100 cells per slide in triplicate for each sample automatically calculated by the cometscan analysis plug-in for the Zeiss MetaCyte software.

### Field emission scanning electron microscopy

Cells were grown on ethanol-sterilised 5 mm × 5 mm silicon chips (Agar Scientific Ltd) in 6-well plates, each well contained 2 silicon chips and was seeded with ~ 0.5 to 1 × 10^5^ cells. Since the Y259X cells did not grow well on plain silicon chips, the chips used for this cell line were coated with 0.01 mg/ml poly-d-lysine (Sigma) overnight and then were left to dry for 2 h before use. The chips were removed at different stages throughout both extraction procedures and then placed and stored in fixative (3% (v/v) glutaraldehyde, 1% (w/v) PFA in 0.1 M sodium cacodylate buffer). The chips were washed in 0.2 M sodium cacodylate buffer and incubated in 1% (w/v) osmium tetroxide (OsO_4_) for 15 min at room temperature. Following this, the chips were placed in distilled water and taken straight-through an ethanol row of 50, 70, 95 and 100% (v/v). Specimens were critical point dried with CO_2_ using a Bal-Tec CPD 030 (BAL-TEC) machine. A Cressington Coating System 308R (Ted Pella, Inc) was then used to sputter coat the chips with either platinum or chromium (1–3 nm). The specimens were viewed using a Hitachi Model S-5200 feSEM (Hitachi High-Technologies) at various accelerating voltages.

## Results

### Which drug treatments restore chromosome territory positions in HGPS fibroblasts?

**Fig. 1:**
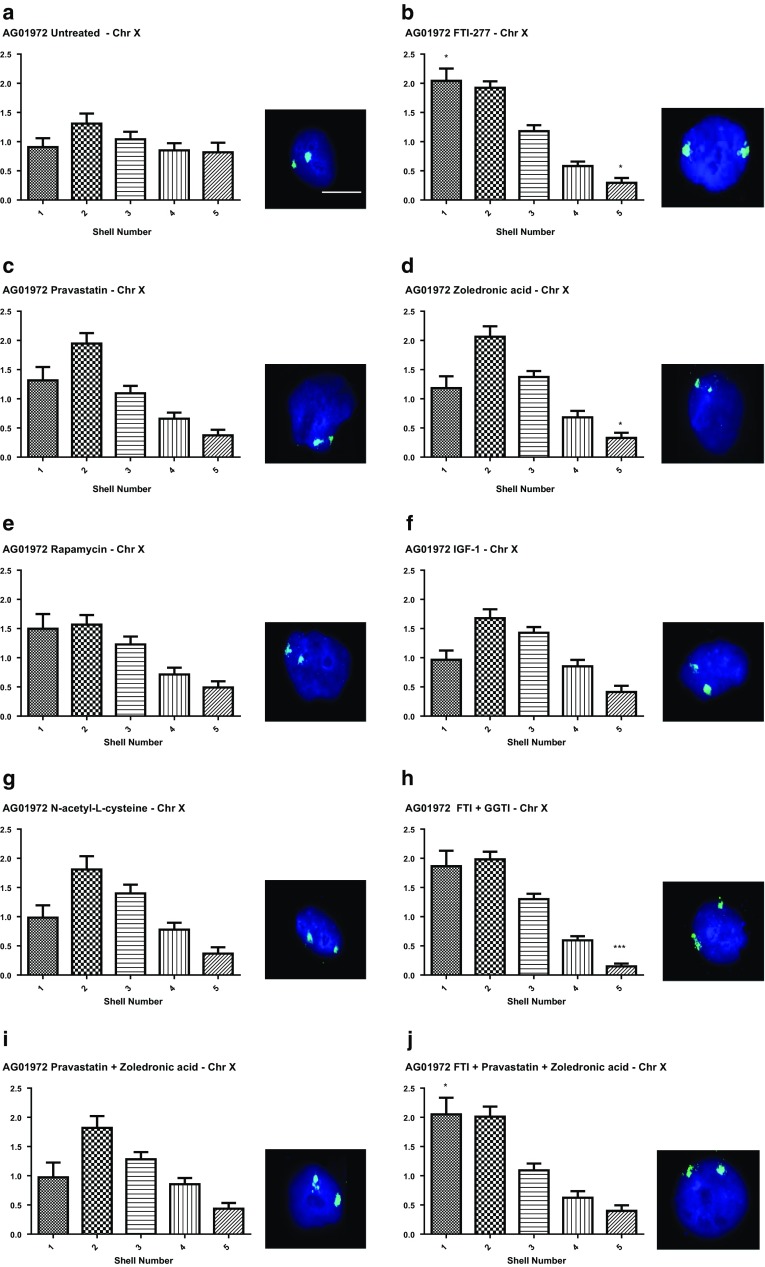
Position of chromosome X within HGPS fibroblast nuclei before and after drug treatments. HGPS fibroblasts, treated and untreated were fixed and subjected to FISH using whole chromosome arm painting probes specific for chromosome X. Images were captured at ×100 magnification. Positional information for the chromosome X territories within interphase nuclei was ascertained through erosion analysis revealing the proportion of chromosome signal (%), normalised by the DAPI signal, over five concentric shells of equal area, starting at the nuclear periphery (shell 1) to the nuclear interior (shell 2). These data are displayed graphically as bar charts with the x-axis displaying the erosion shells from 1 to 5 (left to right. The y-axis displays % normalised chromosome signal. Panels are as follows **a** untreated, **b** FTI-277, **c** pravastatin, **d** zoledronic acid, **e** rapamycin, **f** IGF-1, **g** *N*-acetyl-l-cysteine, **h** FTI-277 and GGTI-2133, **i** pravastatin and zoledronic acid and **j** FTI-277, pravastatin and zoledronic acid. Representative images the interphase nuclei are displayed to the right of each graph. DAPI (blue) is used as a counterstain to delineate the nuclei and the chromosome 18 territories are revealed in green. Scale bar = 10 µM

In previous studies we have shown that chromosome 18, located at the nuclear periphery in normal control proliferating fibroblasts, is mis-localised to the nuclear interior in four different proliferating HGPS cell lines (Meaburn et al. 2007; Mehta et al. 2010a, 2011). Our chromosome positioning is analysed in flattened fibroblast nuclei using 2D-FISH, whole chromosome painting probes and a bespoke erosion analysis (Croft et al. 1999; Clements et al. 2016). The erosion analysis script delineates the imaged nuclei and then erodes in creating five shells of equal area, with shell 1 being the most peripheral and shell 5 the most internal. The intensity of the signal from the DNA stained with DAPI and the chromosome is recorded, the data are normalised by dividing the chromosomal signal by the DNA signal for each shell in each nucleus. These data are plotted as bars to visualise the distribution of chromosomal signal in an average of 50 nuclei. Since we have shown that small chromosome territories such as 18 are repositioned to the nuclear interior in non-proliferating cells (Bridger et al. 2000; Mehta et al. 2007, 2010a) it is imperative to combine the FISH with a marker for proliferation, such as staining for the nucleolar antigen Ki67. Thus this positioning assay of chromosome location, combined with Ki67, permits us to determine if the different drug regimens we have selected can restore genome organisation in proliferating HGPS cells. X chromosome territories provide a control for a chromosome that does not change location in HGPS cells (Meaburn et al. 2007; Mehta et al. 2010a, 2011). Figure 1 displays representative images for X chromosome positioning within classical HGPS fibroblasts treated with the nine different drug treatments. Upon using the erosion analysis for proliferating HGPS cells only (i.e. Ki67 positive) we found that indeed as in our other studies chromosome X territories were predominantly found at the periphery of the nuclei in all situations to varying degrees (Fig. 1a–h), with maximum fluorescent chromosome signal in both shells 1 and 2. The only treatments that enhanced the skew of the graphs more towards shell 1 (i.e. the nuclear periphery) significantly were drug treatments containing FTI i.e. FTI alone (Fig. 1b), FTI and GGTI (Fig. 1h) and FTI with pravastatin and zoledronic acid (Fig. 1j).

**Fig. 2:**
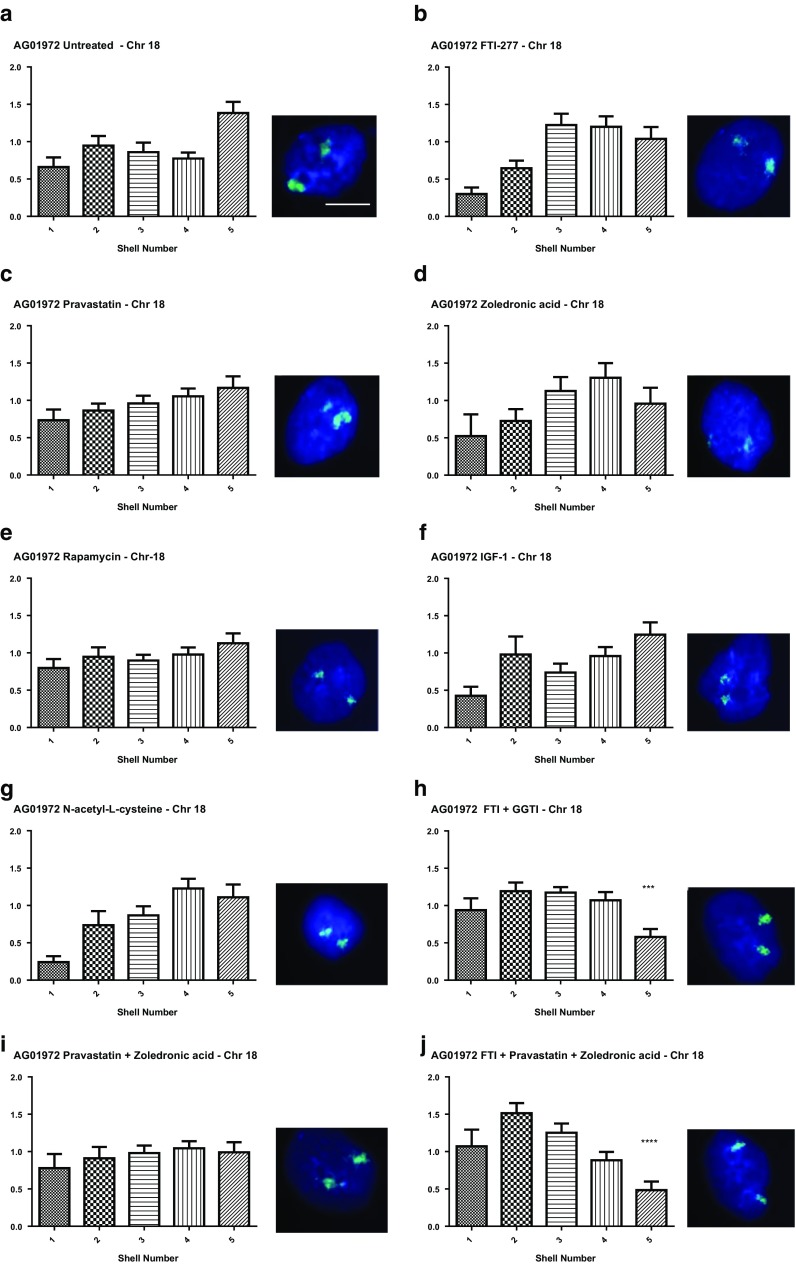
Position of chromosome 18 within HGPS fibroblast nuclei before and after drug treatments. HGPS fibroblasts, treated and untreated were fixed and subjected to FISH using whole chromosome arm painting probes specific for chromosome 18. Images were captured at ×100 magnification. Positional information of the chromosome 18 territories within interphase nuclei was ascertained through erosion analysis revealing the proportion of chromosome signal (%), normalised by the DAPI signal, over five concentric shells of equal area, starting at the nuclear periphery (shell 1) to the nuclear interior (shell 2). These data are displayed graphically as bar charts with the x-axis displaying the erosion shells from 1 to 5 (left to right. The y-axis displays % normalised chromosome signal. Panels are as follows **a** untreated, **b** FTI-277, **c** pravastatin, **d** zoledronic acid, **e** rapamycin, **f** IGF-1, **g** *N*-acetyl-l-cysteine, **h** FTI-277 and GGTI-2133, **i** pravastatin and zoledronic acid and **j** FTI-277, pravastatin and zoledronic acid. Representative images the interphase nuclei are displayed to the right of each graph. DAPI (blue) is used as a counterstain to delineate the nuclei and the chromosome 18 territories are revealed in green. Scale bar = 10 µM

Figure 2 reveals the normalised distribution of the fluorescent signal for chromosome 18 territories in the treated and untreated HGPS cells. Most interestingly we have confirmed our findings from before that FTI is the most successful drug when looking to correct chromosome positioning. Again only drug treatments containing FTI resulted in a significant reorganisation of the genome, as evidenced by the repositioning of the chromosome 18 territories towards the nuclear periphery. These were FTI-277 + GGTI-2133 (Fig. 2h) and FTI-277 + pravastatin + zoledronic acid (Fig. 2j). FTI alone shifted the distribution of the chromosome territories towards the nuclear periphery but was not significant at the 99.9% confidence interval (Fig. 2b), whereas it has been in our other study (Mehta et al. 2011). All other drug treatments i.e. pravastatin alone (Fig. 2c), zoledronic acid alone (Fig. 2d), rapamycin (Fig. 2e), IGF-1 (Fig. 2f) and NAC (Fig. 2g) did not induce any repositioning of chromosome 18 back to the nuclear periphery. Thus it seems that the combinatorial drug treatments containing FTI are the most effective at restoring genome organisation. Given that neither exposure to pravastatin nor zoledronic acid treatments have restored normal chromosome positioning it has to be FTI that is the active component, perhaps allowing an unfarnesylated progerin to make an appropriate lamina connection, presumably through LADs.

### Using DNA halo preparations to analyse alterations in genome interaction with nuclear structure before and after drug treatments in HGPS cells

**Fig. 3:**
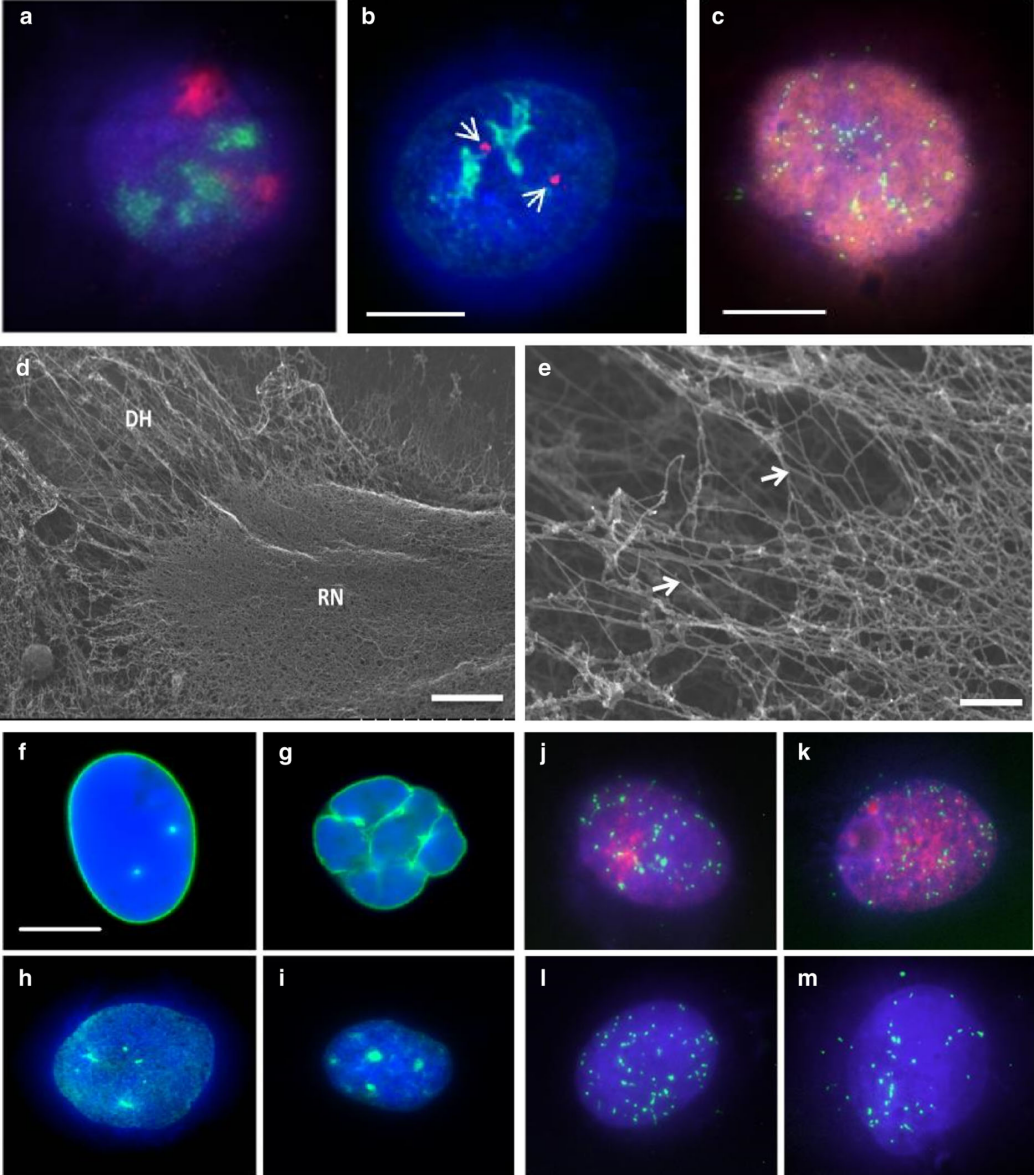
A range of images displaying characteristics of DNA halo preparations. Nucleoskeleton-associated DNA is anchored to the residual nucleus, while extracted, non-associated DNA forms the faint halo surrounding the structure. DNA has been stained using DAPI and can be seen in blue. Panel **a** displays a representative DNA Halo preparation that has been subjected to immuno-FISH using a painting probe for chromosome 17 (red) and the proliferation nucleolar marker pKi67 (green). Panel **b** displays another DNA Halo preparation with again the nucleolar proliferation marker pKi67 in green and *CCND1* gene loci in red. Panel **c** is a DNA Halo preparation displaying a proliferating cell that has incorporated bromo-deoxy-uridine during DNA replication (red), DNA in blue and telomere sequences revealed with a PNA-telo probe in green. Panel **d** shows a residual nucleus surrounded by a halo of DNA visualised by feSEM. The morphology of the residual nucleus (RN) and surrounding DNA halo (DH) Magnification = 6 k scale bar = 2.5 µm. Panel **e** displays extracted, supercoiled DNA (white arrowheads) within the DNA halo, scale bar 300 nm. Panels **f** and **g** are representative control and HGPS fibroblasts with lamin A revealed by indirect immunofluorescence in unextracted cells, respectively and lamin A distribution present in residual nuclei of DNA halo preparations in extracted cells (**h** and **i**). Panels **j**–**m** display representative images of DNA halo preparations with telomeres in green in proliferating control and HGPS fibroblasts (**j** and **k**, respectively) revealed with anti-Ki67 staining (red) and senescent control and HGPS fibroblasts (**l** and **m**, respectively). Note in the HGPS cells telomere signals are located out with the residual nuclei. Scale bars in panels **b** and f = 10 μm

DNA halo preparations have been used for some time now to study interactions of the genome with the nucleoskeleton (Wilson et al. 2016). They are prepared from cells grown on glass, permeabilised with detergent and digitonin and extracted with high salt DNA halo extraction buffer. The idea being that DNA not attached to nuclear structures will extrude beyond the nuclear envelope out of the permeabilsation holes and form a halo of DNA around a residual nucleus. Figure 3 demonstrates DNA halo preparations whereby a range of different analyses can be performed to analyse the genomes’ relationship with the nucleoskeleton within the residual nuclei which is the DNA that is tightly bound to nucleoskeleton. It is possible to visualise whole chromosomes (Fig. 3a) and individual gene loci (Fig. 3b) and telomeres (Fig. 3c, j–m) when performing Halo-FISH. Indirect immunofluorescence is also possible alone and in combination with FISH. Figure 3b, j and k reveal nucleoli within residual nuclei as delineated by anti-Ki67 antibodies. When DNA halo preparations are subjected to feSEM, it is possible to visualise the residual nuclei (Fig. 3d) and the surrounding DNA halo (Fig. 3e), very clearly. It is also possible to use the DNA halo preparations to ask specific and quantifiable questions about nuclear structure and an interacting genome relationship. Figure 3h and i display anti-A-type lamin staining in a control and an HGPS nucleus, respectively. After extraction in the DNA halo preparation some of the A-type lamins have been extracted from the nuclear envelope, leaving/revealing an internal veil of A-type lamins (Fig. 3h, i). Gerdes and colleagues demonstrated that although transcriptionally inactive sequences have loose interactions with the nucleoskeleton, transcriptionally active DNA has a much tighter connection with the nucleoskeleton (Gerdes et al. 1994). Similar results were revealed by Croft et al. that gene-dense human chromosome 19 was much more tightly associated with the nucleoskeleton than gene-poor human chromosome 18 (Croft et al. 1999).

Performing FISH on these preparations, named HALO-FISH using telomere specific probes can delineate specific DNA sequences (Volpi and Bridger 2008; de Lange 1992). It is believed that the association of telomeres with the nucleoskeleton is disrupted in HGPS (Elcock and Bridger 2010). Figure 3j and k reveal telomere distribution in DNA halos of proliferating (Ki67 positive) control and HGPS cells, respectively. Figure 3l and m display the telomere distribution within senescent control and HGPS nuclei, respectively. In the HGPS cells it is noticeable that there are more telomeres (green foci) away from the residual nuclei in the surrounding DNA halos (Fig. 3k and m). Indeed, it was demonstrated that an interaction between a telomere-binding protein, TRF-2 and lamin A/C is disrupted in HGPS which gives rise to telomere instability (Wood et al. 2014).

**Fig. 4:**
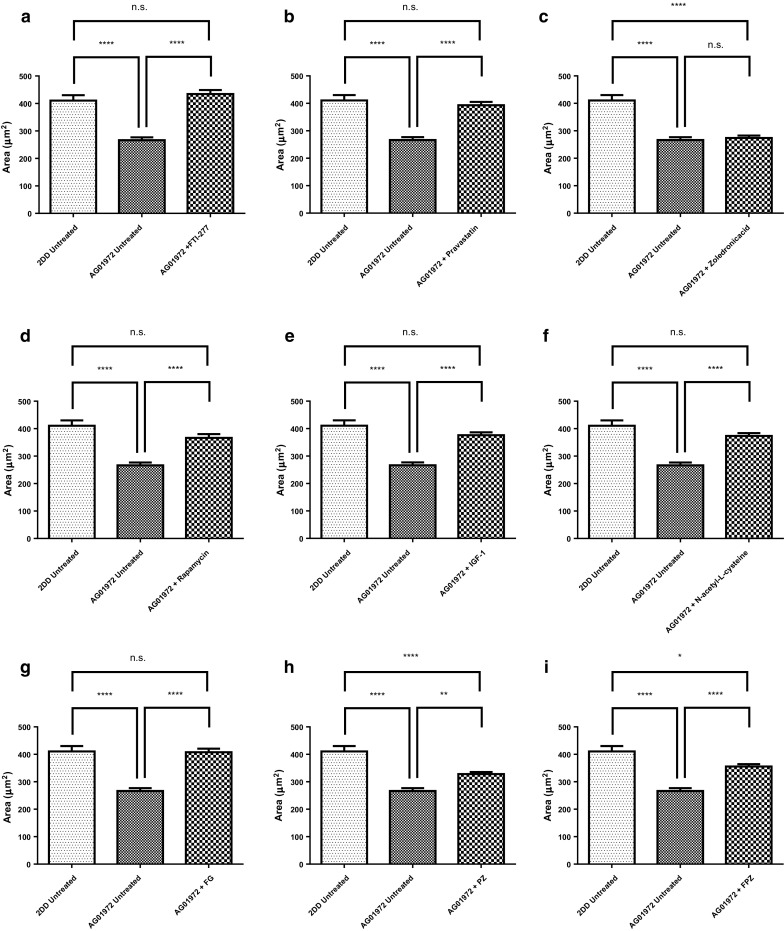
Alterations in size of residual nuclei in DNA Halo preparations of HGPS fibroblasts before and after drug treatments. Images of fixed HGPS DNA halos stained with DAPI taken at ×100 magnification were analysed by ImageJ delineating the residual nuclei and the entire DNA halo. The data displayed in this figure reveals the area measurement of the residual nuclei (x-axis). Results for the treatments are **a** FTI-277, **b** pravastatin, **c** zoledronic acid, **d** rapamycin, **e** IGF-1, **f** *N*-acetyl-l-cysteine, **g** FTI-277 and GGTI-2133, **h** pravastatin and zoledronic acid and **i** FTI-277, pravastatin and zoledronic acid. Error bars represent ± SEM

To date, there are no studies looking at the interactions between the genome and the nucleoskeleton in HGPS in the context of proposed drug treatments. To address this gap of knowledge, this investigation utilises the DNA halo assay to evaluate the efficacy of these drugs in restoring these interactions. Thus, we have perfected DNA halo extractions, combined with FISH, to analyse the association of the nucleoskeleton with the specific regions of the genome (Elcock and Bridger 2010). A high salt DNA halo extraction buffer is used to release unbound DNA from cell nuclei. After fixation 2D-FISH is performed to reveal specific genomic sequences (Elcock and Bridger 2010; Clements et al. 2016). Using simple image analysis through ImageJ FISH signals can be assigned to the residual nucleus i.e. they are bound to the nucleoskeleton and are not extractable or they are located in the nuclear halo and they are extracted and therefore not bound tightly to the nucleoskeleton. Averaged measurement of the area of the residual nuclei in untreated 2DD normal fibroblasts (Fig. 4a) was found to be 410.5 ± 19.7 µm^2^ (n = 50). However in the AG01972 HGPS fibroblasts, the averaged residual nuclear area was significantly reduced to half at 266.4 ± 10.4 µm^2^ (n = 50, p < 0.0001) (Fig. 4b). Strikingly, most of the drug treatments were found to increase the residual nuclear size (Fig. 4, Supplementary Table 1) to be statistically similar to the normal control cells, apart from any treatment containing zoledronic acid (Fig. 4c, h and i, Supplementary Table 1). Moreover, upon treatment of zoledronic acid in 2DD cells, the size of the averaged residual nuclei significantly decreased (p < 0.001) (Supplementary Fig. 1). This result suggests that zoledronic acid may have a detrimental effect on the genome and its ability to bind the nucleoskeleton in HGPS and 2DD fibroblasts or it becomes broken releasing more DNA from the DNA halos.

### The proportion of telomeres remaining within the residual nucleus after a DNA halo preparation

**Fig. 5:**
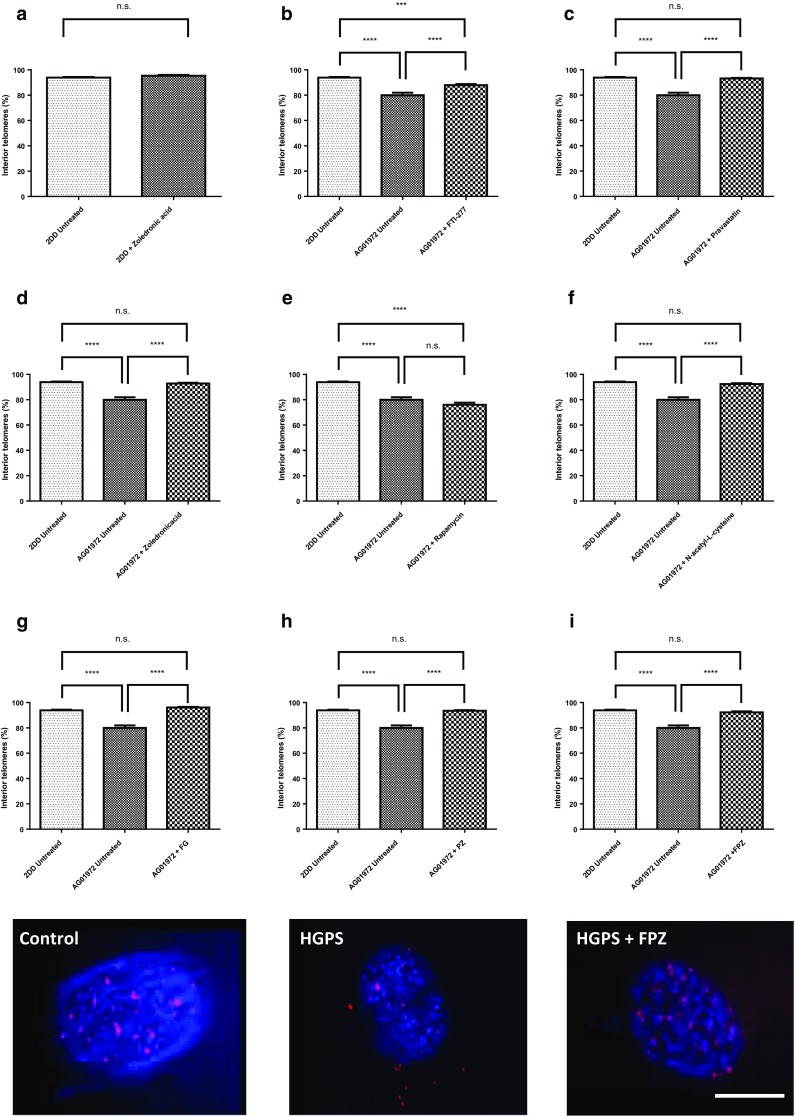
Comparisons of the fraction of telomeres found in the residual nuclei of DNA Halo preparations of HGPS before and after drug treatments. Images of fixed control and HGPS DNA halos stained with both DAPI (blue) and Cy3 (red) telomeric PNA probes were captured at ×100 magnification. Residual nuclei were delineated and the fraction of telomeres determined inside and outside of the residual nuclei. Results for the treatments are shown as **a** 2DD with zoledronic acid, **b** FTI-277, **c** pravastatin, **d** zoledronic acid, **e** rapamycin, **f** *N*-acetyl-l-cysteine, **g** FTI-277 and GGTI-2133, **h** pravastatin and zoledronic acid and **i** FTI-277, pravastatin and zoledronic acid. The proportion of interior telomeres is expressed as a percentage of the total number of telomeres present. Error bars represent ± SEM. Scale bar = 10 µM

Figure 5 displays the data for telomere location within control primary (Fig. 5a) and HGPS (Fig. 5b) fibroblasts halo preparations before and after drug treatments. The telomeres are revealed using a commercial PNA-telo probe that hybridises to telomere repeats. The representative images show the difference between the telomere locations for the HGPS cell line compared to the control cell line. Most telomeres are located within the residual nucleus in the control fibroblasts subjected to extraction. This is in agreement with other studies that conclude that all telomeres should be, and on the whole are, attached tightly to structures within the nucleus. However, in the HGPS cells many of the telomere signals (20%) are found outside of the residual nucleus in the surrounding DNA halo. The actual mean proportion of telomeres remaining within the residual nuclei of 2DD fibroblasts was found to be 93.9 ± 0.5% (Fig. 5a, Supplementary Table 2). However, in AG01972 HGPS fibroblasts, the proportion of interior telomeres was found only to be 80.0 ± 2.0%, which is significantly less than the control (p < 0.0001). When the selected drug treatments are used all treatments bring the number of telomeres within the residual nucleus back to a normal level (Supplementary Table 2), except for FTI alone which only partially rescues (Fig. 5c) the mean proportion of interior telomeres to 87.9 ± 1.0% (n = 37), which is significantly greater than untreated AG01972 fibroblasts (P < 0.0001) but is still significantly different from the normal control cells (P < 0.001). Rapamycin treatment however, reduces the percentage of telomeres within the residual nucleus still further to 76%, but is not found to be significantly different (P < 0.0001) from the untreated HGPS cells.

### DNA damage and drug treatments in HGPS

**Fig. 6:**
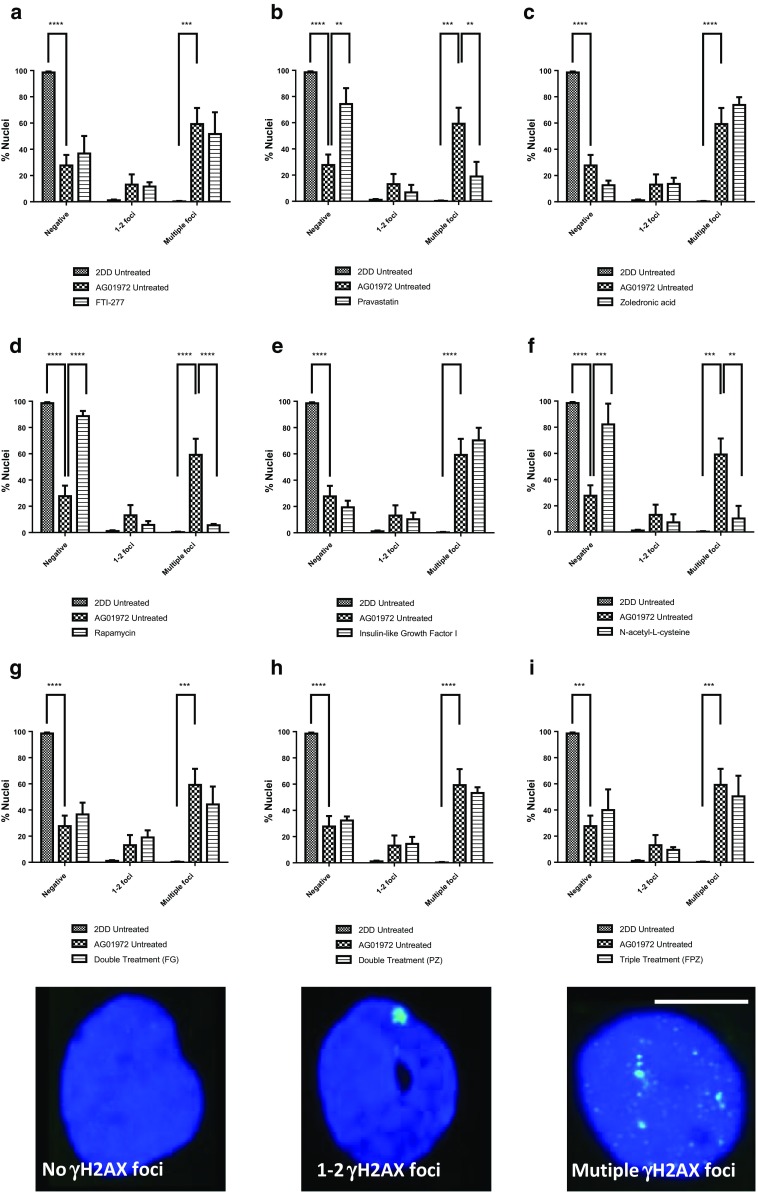
Comparison of the fraction of HGPS fibroblasts displaying DNA damage γH2AX foci before and after drug treatments. Fixed control and HGPS cells (untreated and treated) were subjected to indirect immunofluorescence with a primary antibody recognising phosphorylated histone H2AX a DNA damage marker (green). Distributions of foci were placed in three patterns negative, 1–2 foci and multiple foci and percentages plotted as bar charts **a** FTI-277, **b** pravastatin, **c** zoledronic acid, **d** rapamycin, **e** IGF-1, **f** *N*-acetyl-l-cysteine, **g** FTI-277 and GGTI-2133, **h** pravastatin and zoledronic acid and **i** FTI-277, pravastatin and zoledronic acid. Error bars represent ± SEM. Scale bar = 10 μm

HGPS cells display DNA damage foci consistently (Liu et al. 2005). The effect of specific compounds on the presence of DNA damage in human HGPS fibroblasts has only been performed previously with FTIs (Liu et al. 2006; Constantinescu et al. 2010) and *N*-acetyl-l-cysteine (Richards et al. 2011) within the panel of drug treatments we have selected. Figure 6 displays representative images of the fraction of cells that contain no, 1–2 or multiple γH2AX foci. The results reveal that untreated HGPS fibroblasts exhibit a significantly higher proportion of multiple foci (59.2 ± 12.2%) and a significantly lower proportion of negative staining (no foci) (27.7 ± 8.0%) compared to untreated 2DD control fibroblasts (0.3 ± 0.3%, P ≤ 0.0001 and 98.6 ± 0.7%, n = 3, P ≤ 0.0001 respectively) (Fig. 6). The effect on the presence of DNA damage foci is dramatically different with the different drug treatments. FTI shows no statistical change. On the other hand pravastatin treatment reveals a significant reduction in the fraction of cells displaying multiple foci (18.9 ± 11.2%, n = 3) as well as a significantly higher proportion of cells displaying negative staining (74.3 ± 12.2%) compared to untreated HGPS fibroblasts (Fig. 6b). Interestingly, the other drug that interferes with the mevalonate pathway, zoledronic acid, shows a trend towards an increase in the number of cells displaying multiple γH2AX foci (73.8 ± 5.9%, n = 3) and a lower proportion of negative staining (no foci) (12.6 ± 3.6%, n = 3) compared to untreated HGPS fibroblasts, although statistically insignificant at the 99.9% confidence interval (Fig. 6c) but is worrying nonetheless. IGF-1, like zoledronic acid, results in an apparent increase in cells displaying DNA damage but is also not significant. However, both rapamycin and NAC significantly and dramatically reduce the amount of cells displaying γH2AX foci (Fig. 6d). These results for rapamycin and NAC are not unexpected since they have been shown previously to reduce DNA damage in HGPS cells (Chen et al. 2011; Saha et al. 2014; Richards et al. 2011).

The combinatorial drug treatments are interesting since FTI-277 and GGTI-2133 together (Fig. 6g), do not lead to a significant reduction in γH2AX foci. Neither does pravastatin and zoledronic acid together (Fig. 6h) which is not surprising as their actions may cancel each other out since pravastatin performs well and zoledronic acid may induce more damage. Adding FTI to this combination does not have any additional effect on the reduction of γH2AX foci or an increase in negative cells.

**Fig. 7:**
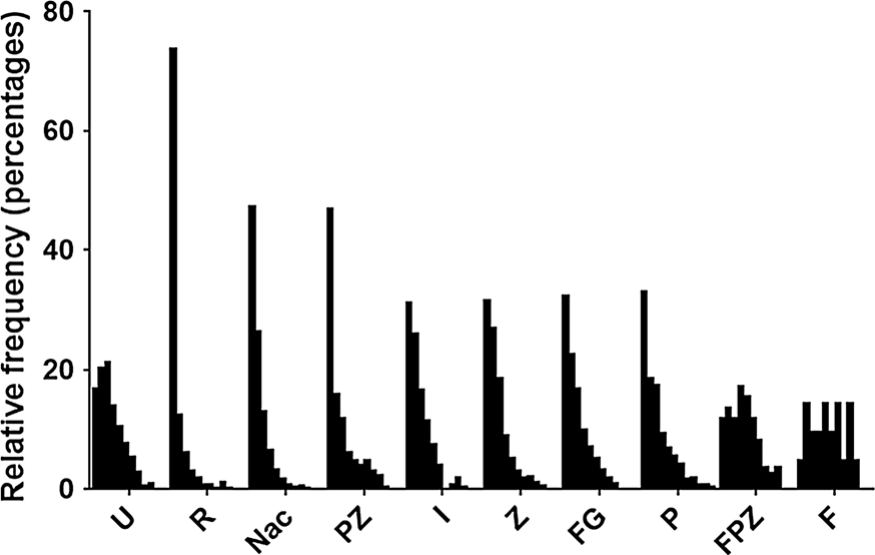
Analysis of DNA damage breaks before and after the nine drug treatments. Cells encapsulated within agarose were treated to alkaline unwinding and denaturation of DNA and then subjected to electrophoresis. Comets were stained with SYBR Gold and analysed using a Zeiss Metafer system running CometScan analysis software. The graph shows relative frequencies of tail moments (%DNA in tail × tail length in μm) for each treatment arranged in order of effectiveness in reducing strand breaks (i.e. small tail moments) compared with the untreated sample. *U* untreated, *R* rapamycin, *NaC N*-acetyl cysteine, *PZ* pravastatin with zoledronic acid, *I* IGF-1, *Z* zoledronic acid, *FG* FTI with GGTI, *P* pravastatin, *FPZ* FTI with pravastatin and zoledronic acid, *F* FTI

We have used, as many other laboratories do, the standard γH2AX indirect immunofluorescence assay and have seen foci in HGPS cells. However, in addition surprisingly we found that zoledronic acid and IGF-1 increase the number of γH2AX foci, although not significantly at the 99.9% CI but the increase was around 20% more cells with DNA damage foci. The γH2AX DNA damage assay is somewhat flawed since it analyses a marker of DNA damage and as such is indirect; thus there is a concern that there still may be DNA damage present that is unmarked by γH2AX or is repairing inefficiently. In order to investigate further actual DNA damage we performed COMET assays that reveal the extent of actual breaks in the cells’ DNA. Cells encapsulated within agarose were treated to alkaline unwinding and denaturation of DNA and subjected to electrophoresis to allow the broken DNA to move through the agarose. Comets were stained with SYBR Gold and analysed using a Zeiss Metafer system running CometScan analysis software. The graph in Fig. 7 displays relative frequencies of Tail Moments (%DNA in tail × tail length in μm) for each treatment arranged in order of effectiveness in reducing strand breaks (i.e. small tail moments) compared with the untreated sample. Most importantly the COMET analyses have revealed that the γH2AX DNA damage foci seen after zoledronic acid and IGF-1 treatment are not actual. This puts doubt over the γ-H2AX assay, especially in HGPS studies and argues that COMET assays should always be performed when analysing the ability of cells to repair their DNA. Rapamycin was by far the best drug at improving the ability of HGPS cells to repair their damaged DNA, with NAC and zoledronic acid in combination with pravastatin also doing well.

## Discussion

The study described here was initiated to test different drugs purported to be of clinical use in a range of genome organisational assays namely—interphase chromosome positioning, DNA halo assays asking questions around genomic attachment to a nucleoskeleton and DNA damage quantification through γH2AX foci and COMET analyses. Mehta et al. (2011) demonstrated that chromosome 18 is located in an interior position in interphase nuclei of senescent HGPS cells and it was revealed that after treatment with FTI-277 alone and together with GGTI-2147, chromosome 18 was repositioned from an internal location to a peripheral one (Mehta et al. 2011). We also demonstrated in this present study that chromosome 18 occupies an interior location in proliferating untreated HGPS nuclei. Using a combinations of drugs, FTI-277 + GGTI-2133 and FTI-277 + pravastatin + zoledronic acid, resulted in relocation of chromosome 18 from nuclear interior to nuclear periphery within Ki-67 positive interphase HGPS nuclei. We have demonstrated before that chromosome X territories remain at the nuclear periphery in all proliferating laminopathy cells analysed (Meaburn et al. 2007). Mehta et al. (2011) also demonstrated that chromosome X localizes to the nuclear periphery in HGPS cells and it was shown that the X chromosome did not change nuclear position after FTI-277 treatment alone nor with FTI-277 and GGTI-2147 together (Mehta et al. 2011). Here in this study we further demonstrated that chromosome X occupies a peripheral location in a further proliferating untreated HGPS cell line. Using combinations of drugs, FTI-277 + pravastatin + zoledronic acid and FTI-277 together with GGTI-2133 seem to result in a relocation of chromosome X more towards the nuclear periphery within proliferating cells but this was insignificant. Interestingly, treatment of HGPS cells with an FTI only showed that chromosome 18 moved towards intermediate location, suggesting GGTI is needed to remove geranyl moieties from the lamin. From these experiments we would suggest that any treatment containing FTI for HGPS will lead to a positive reorganisation of the genome in interphase nuclei. This is reassuring since the latest active drug trial for HGPS children contains FTI (Lonafarnib) in combination with the rapalogue Everolimus (https://clinicaltrials.gov/ct2/show/NCT02579044).

Also from this study, it is clear that FTI-277, pravastatin, rapamycin, IGF-1 and all three combinatorial treatments improve the ratio of total area of DNA halo to residual nucleus suggesting that the amount of DNA within the nucleus, attached to structure within it has been improved by these drug treatments.

There has to be a question mark over γH2AX foci assays since DNA damage highlighted in the DNA damage foci assay (and indirectly from DNA halos) appears not be to be real since when employing the COMET assay there was no DNA damage apparent. This was especially concerning for the zoledronic acid treatment. However, we would suggest that consideration be taken with any treatments using zoledronic acid since it is used as a cancer treatment to arrest proliferating cells through DNA damage response and we are not the first to see DNA damage foci in cells treated with it (Iguchi et al. 2007; Ohnuki et al. 2012) and a further, deeper investigation of the potential of zoledronic acid to promote DNA damage is called for although there is a study which implies zoledronic acid attenuates DNA damage (Misra et al. 2015).

Rapamycin is the other drug being used in combination with FTI in the new clinical trial for HGPS. In this study rapamycin was the best drug for permitting DNA damage in the HGPS cells to be repaired. This means that the two top performing drugs that we have found in our panel of nine treatments with four assays are being given to children with HGPS. The only point of concern could be that rapamycin impacts on the interaction of telomeres with the nucleoskeleton—this also warrants further investigation. However, it has been suggested that analogues of rapamycin such as temsirolimus might substitute for rapamycin given that there are some potential drawbacks to using rapamycin (Gabriel et al. 2016).

### Potential drugs for treatment of HGPS

Since the original studies leading to the choices of FTI, Pravastatin, Zoledronic Acid and Rapamycin, other drugs have come to the fore as potential treatments for HGPS. These include drugs such as the anti-oxidant sulforaphane and the proteasome inhibitor MG132 which both encourage autophagy, helping to clear the toxic progerin from HGPS cells (Gabriel et al. 2017; Harhouri et al. 2017). Another combinatorial treatment with all-trans retinoic acid, another inducer of autophagy, in combination with rapamycin normalises HGPS cells by reducing progerin, farnesylated pre-lamin A, and  DNA damage foci (Pellegrini et al. 2015).

The anti-diabetic drug metformin is also hailed as a treatment for HGPS as well as an anti-ageing treatment—given that diabetic patients on metformin live longer than the control group (Bannister et al. 2014). Metformin treatment of HGPS cells results in the down-regulation of progerin expression (Egesipe et al. 2016; Park and Shin 2017). Methylene blue is another anti-oxidant and potential anti-ageing product that seems to have great potential in treating HGPS (Xiong et al. 2017). Other successful compounds that reduce reactive oxygen species in HGPS include the rho-associated protein kinase (ROCK) inhibitor—Y-27632, reducing DNA damage, improving nuclear shape and restoring mitochondrial function (Kang et al. 2017).

Interestingly, a compound called NAT10 or Remodelin (Larrieu et al. 2014) also improves nuclear morphology in HGPS cells, as well as a reduction in γH2AX foci but also improves chromatin organisation as assessed by staining with anti-H3K9me3 antibodies which is in common with the study using all-trans retinoic acid and rapamycin (Pellegrini et al. 2015). Nucleolar structure is also improved, as has been shown for FTI as well (Mehta et al. 2010b).

Most excitingly, new drug possibilities have been found that could substitute for FTIs, given that two mono-amino pyrimidines inhibit farnesylation by inhibiting farnesyl pyrophosphate synthase and farnesyl transferase (Blondel et al. 2016). Vitamin D receptor has been found to be linked to premature ageing (Keisala et al. 2009), and using 1a, 25-dihydroxyvitamin D3 improves a range of HGPS phenotypes (Kreienkamp et al. 2016).

### Telomere anchorage by the NM in normal and HGPS fibroblasts before and after drug treatment in the DNA halo assay

Telomeres are shortened with each cell division due to the end replication problem, once telomere length reaches a critical length, the cells enter replicative senescence (Stewart and Weinberg 2006). It has been demonstrated that such eroded telomeres trigger DNA damage checkpoint responses which in turn leads to this irreversible G1/S cell cycle arrest (di Fagagna et al. 2003). In light of these facts, we thought it pertinent to examine and compare telomere interactions with the nucleoskeleton in control (2DD) and typical HGPS (AG01972) HDFs by coupling the DNA halo preparation with telomere PNA FISH (Bridger and Lichter 1999), using combination of drug treatments. We have demonstrated that telomere anchorage with the nucleoskeleton in AG01972 HGPS line, which produces a mutant form of lamin A (progerin) is severely perturbed. More than 20% of telomeres were found outside residual nuclei. De Vos et al. (2010) reported that in HDF^mut/mut^ cells, telomere mobility is higher and less confined compared with normal fibroblasts. However, intriguingly in their experiments, it was exhibited that telomere movement appeared more restricted in HGPS cells (De Vos et al. 2010). Remarkably, treatment of HGPS cells with FTI is able to increase telomere mobility (De Vos et al. 2010). It is known that progeria cells have shorter telomeres (Allsopp et al. 1992) and chromosomal aberrations in the setting of telomere dysfunction were shown to be triggered by progerin expression in HGPS cells (Benson et al. 2010). Rippe’s group (2009) showed that shorter telomeres have increased telomere mobility in a telomerase negative human cell line (Jegou et al. 2009) and in line with our findings which has demonstrated that telomere binding to the nucleoskeleton is reduced in HGPS cells. Gonzalez-Suarez et al. (2009) demonstrated that the accumulation of farnesylated lamin A is concomitant with a migration of telomeres towards the nuclear periphery (Gonzalo-Suaraz et al. 2009). Raz et al. (2008) reported that association of telomeric repeats with lamina intranuclear structure is increased in cells expressing lamin mutants (Raz et al. 2008). It can be suggested that for the correct localization of telomeres within the nucleus, intranuclear lamins play a vital role (De Vos et al. 2010). Whereas, telomere-nucleoskeleton interactions in control cell line (2DD) reveal that approximately 93–94% of telomeres were found within residual nuclei. The results are also in agreement with an earlier study which shows that at least 80% of telomeric DNA is attached to this structure (de Lange 1992). The findings also confirm previous HALO-FISH experiments using telomere-specific probes (Luderus et al. 1996). Zoledronic acid treated 2DD cells showed that 95–96% of telomeres were found within the residual nucleus which does not change statistically the telomere location. From this study, it is clear that FTI-277, pravastatin, zoledronic acid, *N*-acetyl-l-cysteine and all three combination treatments, FG, PZ, FPZ, have a positive effect on anchoring the genome to the nucleoskeleton in AG01972 cell line. On the contrary, rapamycin treated AG01972 cells give rise to a reduction of anchorage of telomeres to the nucleoskeleton having approximately 76–77% of telomeres in the residual nuclei but does not show any significant difference compared with untreated AG01972 cells.

In conclusion, the results of this study have potentially far-reaching implications for the treatment of sufferers with HGPS. For instance, of the drugs currently used in clinical trials, only pravastatin led to a reduction in DNA damage, and not when in combination as used in the trials. FTIs do not significantly reduce DNA damage despite having well recognised improvements to nuclear shape. Rapamycin performed well with respect to aiding HGPS cells repair DNA damage, but was not able to restore normal genome organisation. Thus, it could be envisioned that rapamycin could be used in combination with an FTI. From the analyses in this study it is clear that further studies should concentrate more on the effects of drug treatments on other nuclear proteins, genome organisation, and gene expression in HGPS fibroblasts rather than just assessing nuclear shape.

## Electronic supplementary material

Below is the link to the electronic supplementary material.

Supplementary material 1 (DOCX 13 kb)

Supplementary Fig. 1 Analysis of residual nucleus size, total size of DNA halo and residual nucleus to total area of halo ratio of DNA halo preparations of 2DD fibroblasts with and without zoledronic acid treatment. Images of fixed 2DD DNA halos stained with DAPI taken at ×100 magnification using a Zeiss Axiovert 200M with a Plan-NEOFLUAR ×100/1.30 objective lens (Carl Zeiss, Germany) and high resolution digital camera (AxioCam b/w, Carl Zeiss, Germany) under the control of AxioVision software (Carl Zeiss). Results for the treatment of zoledronic acid are shown as a) residual nucleus, b) total DNA halo size and c) residual nucleus to total area of halo ratio. Error bars represent ± SEM. Supplementary material 2 (DOCX 437 kb)

## Abbreviations

DSB: Double stand break
FTI: Farnesyltransferase inhibitor
GGTI: Geranylgeranyltransferase inhibitor
GH: Growth hormone
HGPS: Hutchinson–Gilford progeria syndrome
ICMT: Isoprenyl-cysteine-carboxy-methyltransferase
IGF-1: Insulin-like growth factor 1
mTOR: Mammalian target of rapamycin
ROS: Reactive oxygen species
