# Supplementary material for: Farnesyltransferase inhibitor and rapamycin correct aberrant genome organisation and decrease DNA damage respectively, in Hutchinson–Gilford progeria syndrome fibroblasts
Source: Biogerontology. 2018 Jun 15;19(6):579–602. doi: 10.1007/s10522-018-9758-4 (PMC6223735; doi:10.1007/s10522-018-9758-4)
Supplement: Supplementary file 1 — Supplementary material 1 (DOCX 13 kb) [file 10522_2018_9758_MOESM1_ESM.docx]

**Supplementary Table 1**

| Cell Type | Residual Nuclear Size in μm^2^ | Significantly different to 2DD control. |
| --- | --- | --- |
| 2DD | 410.5 ± 19.7 |  |
| AG01972 | 266.4 ± 10.4 | P<0.0001 |
| AG01972 + FTI | 434.5 ± 19.7 |  |
| AG01972 + PA | 393.1 ± 12.2 |  |
| AG01972 + RAP | 366.6 ± 13.8 |  |
| AG01972 + ZA | 274.1 ± 8.5 | P<0.0001 |
| AG01972 + IGF-1 | 375.8 ± 10.7 |  |
| AG01972 + NAC | 373.5 ± 10.4 |  |
| AG01972 + FTI+GGTI | 407.6 ± 13 |  |
| AG01972 + PA+ZA | 328.1 ± 7.6 | P<0.0001 |
| AG01972 + FTI+PA+ZA | 355.7 ± 8.7 | P<0.0001 |

# Supplementary Table 2

| Cell Type | % Telomeres remaining in the residual nucleus after a DNA halo extraction | Significantly different to 2DD control. |
| --- | --- | --- |
| 2DD | 93.9 ± 0.5 |  |
| AG01972 | 80 ± 2.0 | P<0.0001 |
| AG01972 + FTI | 87.9 ± 1.0 | P<0.0001 |
| AG01972 + PA | 93.2% ± 0.5 |  |
| AG01972 + RAP | 76 ± 1.0 | P<0.0001 |
| AG01972 + ZA | 92.8 ± 1.0 |  |
| AG01972 + NAC | 92.4 ± 1.0 |  |
| AG01972 + FTI+GGTI | 96.1 ± 0.5 |  |
| AG01972 + PA+ZA | 93.6 ± 0.5 | P<0.0001 |
| AG01972 + FTI+PA+ZA | 92.3 ± 0.8 | P<0.0001 |
